# Antimicrobial peptide expression in the cockroach gut during enterobacterial infection is specific and influenced by type III secretion

**DOI:** 10.1242/bio.059414

**Published:** 2022-05-25

**Authors:** Matthew Turner, Jose E. Pietri

**Affiliations:** University of South Dakota, Sanford School of Medicine, Division of Basic Biomedical Sciences, Vermillion, SD 57069, USA

**Keywords:** German cockroach, Blattella, Salmonella, Infection, Immunity, Antimicrobial peptide

## Abstract

Omnivorous synanthropic cockroaches, such as the German cockroach (*Blattella germanica*), are reservoirs and vectors of enteric bacterial pathogens. A lifestyle conducive to frequent encounters with high loads of diverse bacteria may have led to the evolution of unique innate immune systems in these insects. The innate immune response of insects relies largely on generalized mechanisms to sense and eliminate foreign microbes. However, analyses of the genomes of common synanthropic cockroaches previously revealed a repertoire of pathogen associated molecular pattern (PAMP) receptors and antimicrobial peptides (AMPs) that is significantly expanded relative to most holometabolous insect models and vectors, supporting the intriguing possibility that cockroaches may encode enhanced recognition within their immune system and may possess an enhanced capacity to fine tune innate immune responses. Investigating how cockroaches respond to infection with enterobacteria provides the opportunity to expand our fundamental knowledge of the regulation of insect innate immunity in a context that is biologically and medically relevant. German cockroaches can harbor both *Salmonella enterica* serovar Typhimurium and *Escherichia coli* in their gut without experiencing pathogenesis. The former colonizes the gut and replicates while the latter persists only transiently. We hypothesized that differences in the innate immune response may contribute to or result from the difference in infection dynamics between the two enterobacteria. To test this hypothesis, we used qRT-PCR to analyze expression of five genes encoding representative AMPs (Attacins, Blattellicin, Defensins) in the gut of German cockroaches 1 and 24 h after ingestion of live or heat-killed enterobacteria. We found that robust AMP expression was induced in response to ingestion of a live wild-type strain of *S*. Typhimurium, but not in response to live *E. coli*, heat-killed *S*. Typhimurium, or a live mutant strain of *S*. Typhimurium lacking type III secretion systems. These results indicate that the cockroach immune system does not respond to stimulation with high levels of ingested bacterial PAMPs such as peptidoglycan. Rather, AMP expression in the gut appears to be induced by active bacterial colonization involving type III secretion. We speculate that this form of regulation may have evolved to prevent over activation of the immune system from frequent ingestion of innocuous, non-colonizing, or non-viable bacteria. While additional work is needed to delineate the molecular mechanisms underlying our observations, our findings provide significant novel insight into the immunological adaptation of cockroaches to life in septic environments as well as the factors that regulate bacterial pathogen transmission by these insects.

## Abstract (precis)

**Summary:** This study demonstrates that antimicrobial peptide gene expression in the German cockroach gut can be highly specific, responding to type III secretion-dependent colonization processes rather than to general bacterial associated molecular patterns. This finding provides new insight into the immunological adaptation of cockroaches to life in septic environments as well as the factors that regulate bacterial pathogen transmission by these insects.

## INTRODUCTION

Some synanthropic cockroaches, such as the German cockroach, *Blattella germanica*, can be prolific pests and thrive in diverse environments worldwide. These pests can subsist on a wide range of organic material, adapting their diet to local conditions (McPherson et al., 2021). In polluted or unsanitary environments, cockroaches may routinely consume refuse, spoilage, and human or animal feces (Zurek and Schal, 2004; Graczyk et al., 2005). The gut microbiota of German cockroaches is partially acquired vertically via their diet. As such, the insects typically harbor a diverse array of microbes within their gut (Kakumanu et al., 2018). Human pathogenic bacteria that cause enteric disease (e.g. *Escherichia coli*, *Salmonella* spp.) are routinely detected in association with cockroaches (Nasirian, 2019), and some species, such as *Salmonella enterica* serovar Typhimurium, undergo replication in the cockroach gut after they are ingested (Turner et al., 2021). Cockroaches can act as vectors when they shed these bacteria in their feces, contributing directly to the spread of infections among humans (Graffar and Mertens, 1950). As they are long-lived and experience frequent exposure to high loads of bacteria, particularly via ingestion, German cockroaches must balance efficient elimination of dangerous entomopathogens with costs associated with immune activation to avoid detrimental stimulation of the immune system in response to innocuous or non-viable challenges. Relatedly, the immune system in the gut may be a key driver of vector competence for human pathogens.

Insects rely on an innate immune system to sense and eliminate foreign microorganisms in a largely generalized manner. Over the last several decades, studies in the model organism *Drosophila melanogaster* have elegantly elucidated the contributions and roles of the Toll and IMD signaling pathways to the innate immune response against bacteria (De Gregorio et al., 2002; Lemaitre and Hoffmann, 2007; Kurata, 2014). Importantly, many aspects of Toll and IMD signaling in *D. melanogaster* are now known to be conserved in other insect species. Sensing of Gram-negative bacteria through the IMD pathway relies primarily on the recognition of diaminopimelic acid-type peptidoglycan (DAP-PGN) by peptidoglycan recognition proteins (PGRPs) such as the transmembrane receptor PGRP-LC (Choe et al., 2005). Meanwhile, sensing of most Gram-positive bacteria occurs through the Toll pathway via recognition of lysine-type peptidoglycan (LYS-PGN) by soluble PGRPs such as PGRP-SA (Michel et al., 2001). Binding of LYS-PGN promotes indirect activation of the Toll pathway via cleavage of the ligand Spätzle, which then binds the Toll receptor. Downstream of both pathways, the transcription of genes encoding antimicrobial peptides (AMPs) that function individually or in concert to directly lyse various types of microbes is induced (Lin et al., 2020).

Regulation of the Toll and IMD pathways is complex, and crosstalk has been shown to take place between the two (Nishide et al., 2019). In *D. melanogaster*, homeostatic feedback mechanisms exist to prevent sustained activation of the IMD pathway below certain bacterial densities. These mechanisms involve secreted PGRPs (e.g. PGRP-LB) that scavenge and degrade PGN to prevent receptor binding, as well as inhibitory membrane PGRPs (e.g. PGRP-LF) that can interact with PGRP-LC to prevent signal transduction (Maillet et al., 2008; Paredes et al., 2011). Critically, Toll and IMD mediated AMP responses are active not only systemically in the insect hemolymph but are also important locally in the gut barrier epithelium during oral infection (Liehl et al., 2006). For instance, PGRP-LA and PGRP-LE both regulate AMP expression in epithelial tissue in *D. melanogaster* (Takehana et al., 2004; Gendrin et al., 2013).

Recent *in silico* analyses of the genomes of two synanthropic cockroach species, the American cockroach, *Periplaneta americana*, and the German cockroach, *B. germanica,* revealed an intriguing aspect of their immune systems (Li et al., 2018; Silva et al., 2020). That is, when compared to distantly related holometabolous insects such as fruit flies and mosquitoes, both species encode an expanded arsenal of genes involved in the recognition and elimination of microbes. These include genes encoding pathogen associated molecular pattern (PAMP) receptors, such as PGRPs and Gram-negative bacteria binding proteins (GNBPs), along with AMPs. Within the order Blattodea, many of the same gene groups appear to have subsequently contracted as wood-feeding, eusocial termites diverged evolutionarily from cockroaches (He et al., 2021). The expansion of AMP genes is particularly striking in *B. germanica*. In this species, 39 putative AMP genes have been identified, among which are 16 Defensin-like genes, 13 Drosomycin-like genes, three Termicin like-genes, and seven Attacin-like genes (Silva et al., 2020). Four of the Attacin-like genes identified in *B. germanica* encode proteins that are ∼200 amino acids in length and contain a glutamine/glutamic acid rich central region. These unique AMPs were termed Blattellicins. In line with seminal organismal studies demonstrating specific systemic immune responses in *P. americana* (Faulhaber and Karp, 1992), the expansion of PAMP receptors and AMPs suggests that cockroaches may indeed encode enhanced recognition within their immune system and may possess an enhanced capacity to fine tune innate immune responses. These are unusual possibilities for an insect. Yet, next to nothing is known about how cockroaches recognize and respond to bacterial infections at the molecular level, as functional experiments have not been pursued.

Our recent work examining the colonization of the German cockroach gut by two human pathogenic enterobacteria, *E. coli* and *S.* Typhimurium revealed a strong contrast. These bacteria are fundamentally similar and are both frequently acquired by cockroaches in nature (Nasirian, 2019). However, we found that *S.* Typhimurium actively colonizes and replicates in the cockroach gut, resulting in a stable bacterial load for at least 7 days (Turner et al., 2021). On the other hand, we found no evidence of replication by *E. coli*, which rapidly declined and was mostly eliminated from the cockroach gut within 3 days (Ray et al., 2020). Investigating how cockroaches respond to ingested enterobacteria thus presented the opportunity to advance our fundamental understanding of the regulation of insect immunity in a context that is both biologically and medically relevant (Little et al., 2005; Chambers and Schneider, 2012). We hypothesized that cockroaches may differentially recognize and respond to ingested *S.* Typhimurium and *E. coli* either as a result of or contributing to differences in their colonization dynamics. We further hypothesized that because *S.* Typhimurium requires type III secretion systems for efficient transmission in the cockroach feces (Turner et al., 2021), it may use type III secretion to manipulate the vector immune response and enhance its own transmission. To address these hypotheses, in the present study we utilized qRT-PCR to quantify expression of five AMP genes in the gut of *B. germanica* 1-and-24 h after various enterobacterial stimuli. Specifically, expression of two Attacin genes, a Blattellicin gene, and two Defensin genes was examined after ingestion of live wild-type *S*. Typhimurium, live *E. coli*, heat-killed *S*. Typhimurium, and a live mutant strain of *S*. Typhimurium lacking type III secretion systems.

## RESULTS

### Survivorship of cockroaches fed *S.* Typhimurium

**Fig. 1.**
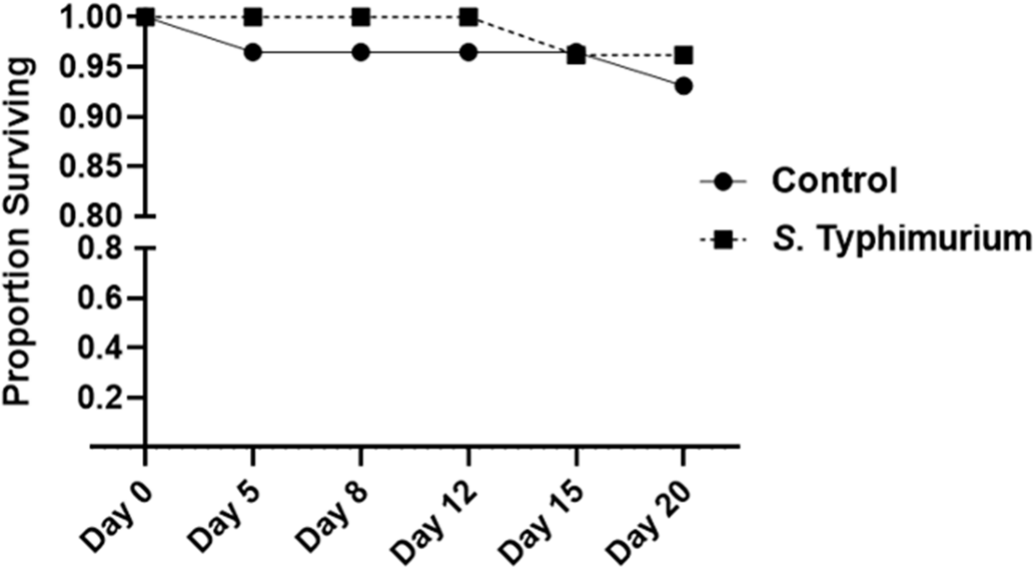
**Survivorship of cockroaches fed live *S.* Typhimurium.** Groups of adult male cockroaches were fed either a culture of *S*. Typhimurium (OD_600_=1) or sterile LB medium as a control. Deaths were monitored periodically for 20 days. The plotted data are derived from a replicate consisting of 29 individuals in the control group and 26 individuals in the infected groups. Results are representative of two independent trials. The data were analyzed by Fisher's exact test.

Survivorship analysis of cockroaches fed *S.* Typhimurium confirmed that this bacterium is not pathogenic to cockroaches even when high levels are ingested (Fig. 1), similar to *E. coli* B21, the other bacterium used in this study (Ray et al., 2020). A consistent pattern was observed in both experimental trials. Average survival 20 days after feeding was 94.7% in cockroach cohorts that consumed *S*. Typhimurium and 83.3% in cockroach cohorts fed sterile LB medium (Fisher's exact test, *P*=0.268).

### Expression of antimicrobial peptide genes in response to live bacteria

AMP gene expression patterns in response to ingestion of live bacteria differed slightly 1-h and 24-h post-feeding, and the scale of AMP induction was generally lower at 24-h post-feeding. More interestingly, the different bacteria that we fed to cockroaches had markedly contrasting effects on gut AMP gene expression relative to baseline at both time points.

**Fig. 2.**
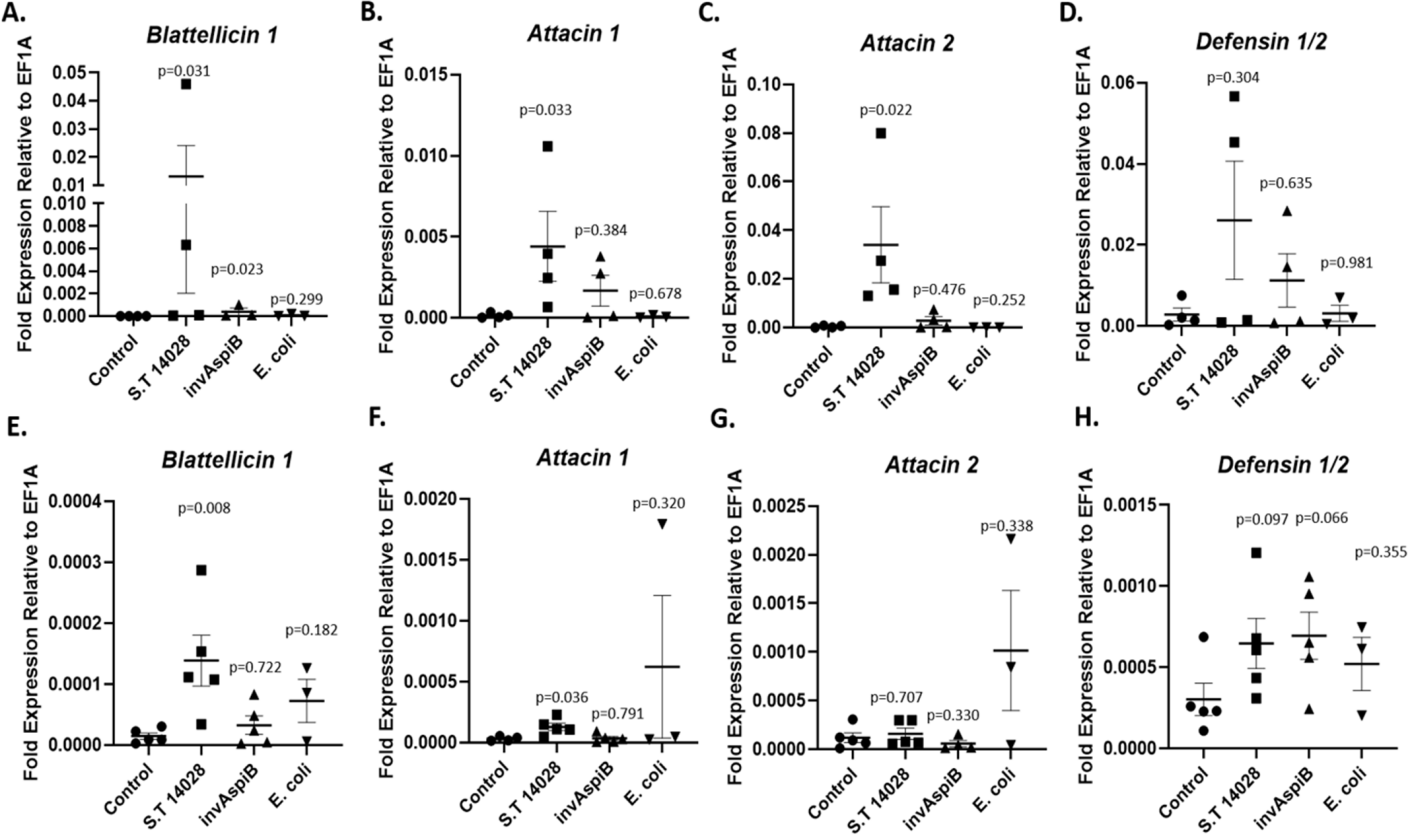
**Antimicrobial peptide gene expression in response to live *S.* Typhimurium (S.T 14028, invAspiB) or *E. coli* in the cockroach gut.** (A–D) 1 h after ingestion. (E–H) 24 h after ingestion. *N*=4–5 cockroaches per treatment per time point. Plotted are the mean expression values relative to the housekeeping gene with standard error (s.e.m.). The *P*-values shown above each column correspond to the comparison made between that column and the control column. Data were analyzed by Kruskal–Wallis one-way ANOVA.

At 1-h post-feeding (Fig. 2A–D), wild-type *S.* Typhimurium (ANOVA, *P*=0.031) and the type III secretion deficient invAspiB mutant (ANOVA, *P*=0.023) both induced expression of Blattellicin 1 relative to control. However, only the wild-type *S.* Typhimurium strain induced expression of Attacin 1 (ANOVA, *P*=0.033) and Attacin 2 (ANOVA, *P*=0.022) at this time point. Meanwhile, expression of Defensin 1/2 was not induced by any of the three bacteria (ANOVA, *P*>0.3 for all). Ingestion of *E. coli* did not induce expression of any of the AMP genes examined 1-h post feeding (ANOVA, *P*>0.25 for all), and expression levels in response to this bacterium were remarkably similar to expression levels in unstimulated controls.

At 24-h post-feeding (Fig. 2E–H), only the wild-type strain of *S.* Typhimurium (ANOVA, *P*=0.008), but not the type III secretion deficient invAspiB mutant (ANOVA, *P*=0.722) induced expression of Blattellicin 1. Similarly, wild-type *S.* Typhimurium induced expression of Attacin 1 (ANOVA, *P*=0.036) but the mutant strain did not (ANOVA, *P*=0.791). Neither strain of *S.* Typhimurium, nor *E. coli*, induced expression of Attacin 2 24-h after ingestion (ANOVA, *P*>0.3 for all), in contrast to results for this gene at the 1-h time point. Furthermore, expression of Defensin 1/2 was not significantly induced by any of the bacteria 24-h after feeding (ANOVA, *P*>0.06 for all). However, it is noteworthy that expression of Defensin 1/2 was consistently higher in cockroaches fed either strain of *S.* Typhimurium than in control cockroaches. While the calculated *P*-values of 0.097 and 0.066 did not reach the conventional level of statistical significance, these findings may have some biological relevance.

### Expression of antimicrobial peptide genes in response to heat-killed Salmonella

**Fig. 3.**
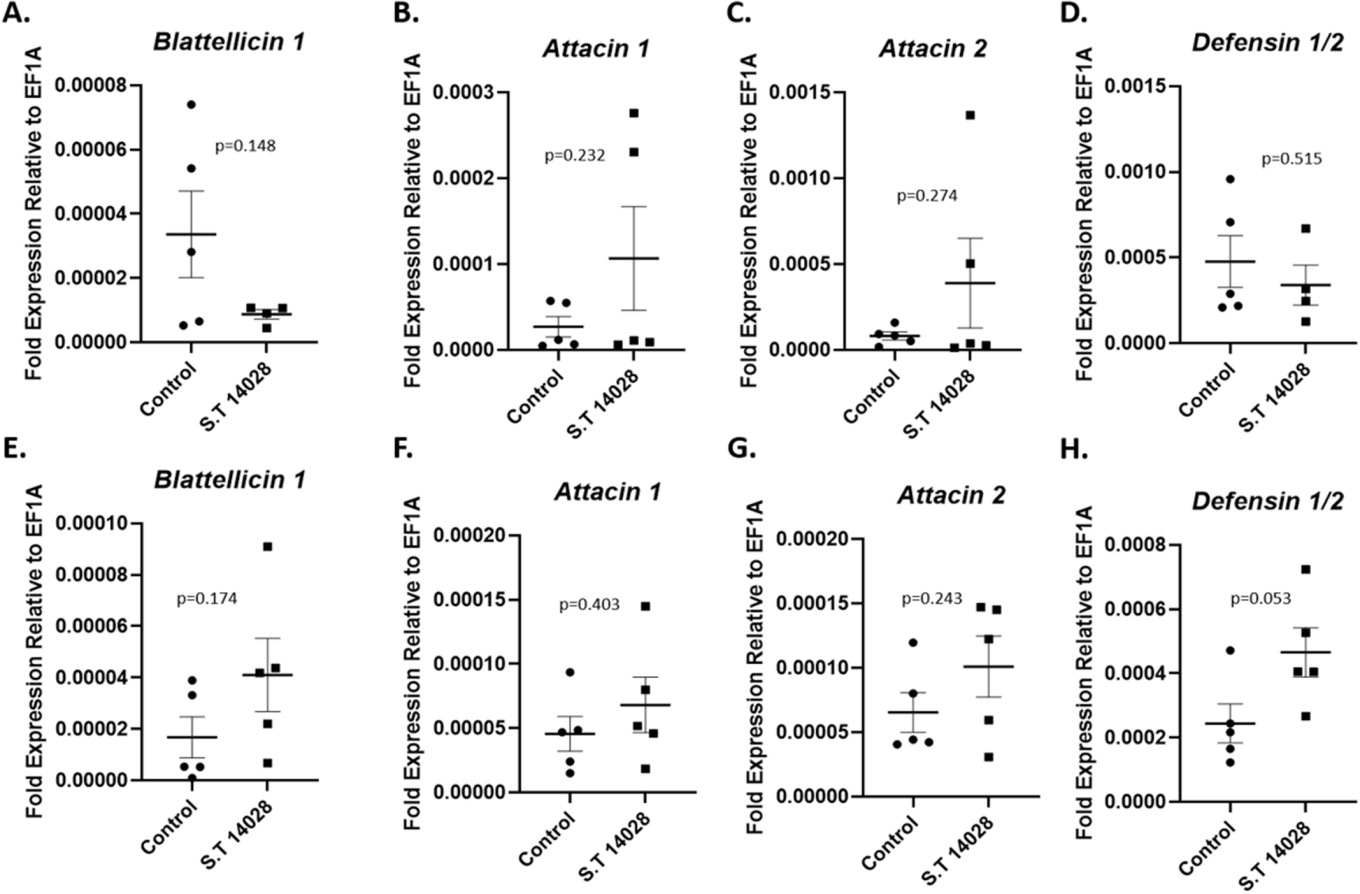
**Antimicrobial peptide gene expression in response to heat-killed *S.* Typhimurium (S.T 14028) in the cockroach gut.** (A–D) 1 h after ingestion. (E–H) 24 h after ingestion. *N*=4–5 cockroaches per treatment per time point. Plotted are the mean expression values relative to the housekeeping gene with standard error (s.e.m.). The *P*-values shown correspond to the results of comparison by unpaired *t*-test.

In order to isolate the effects of bacterial PAMP stimulation on AMP gene expression in the gut, we next fed cockroaches heat-killed wild-type *S.* Typhimurium (Fig. 3). This was necessary as *S*. Typhimurium undergoes replication and other active colonization processes in the cockroach gut (Turner et al., 2021), which could potentially influence the immune response to a live challenge. While live *S.* Typhimurium induced expression of several AMPs (Fig. 2), the same was not true of heat-killed bacteria.

No significant difference in expression of Attacin 1, Attacin 2, Blattellicin 1, or Defensin 1/2 was noted between cockroaches fed heat-killed *S.* Typhimurium and control cockroaches 1 h after feeding (*t*-test, *P*>0.14 for all) (Fig. 3A–D). Similarly, 24 h after feeding, there was no significant difference in expression of Attacin 1, Attacin 2, or Blattellicin 1 (*t*-test, *P*>0.17 for all) (Fig. 3E–H). Intriguingly, expression of Defensin 1/2 24 h after ingestion of heat-killed *S.* Typhimurium was consistently higher than in controls (*t*-test, *P*=0.053). This trend was in line with the effects of live *S.* Typhimurium on Defensin 1/2 expression at the 24-h time point (Fig. 2H). Once again, while the calculated *P*-value did not reach the conventional level of statistical significance, this finding may have some biological relevance, especially when considering that a similar result was obtained after feeding live *S*. Typhimurium.

## DISCUSSION

By extending recent *in silico* characterization of the innate immune repertoire of *B. germanica* (Silva et al., 2020) in a functional direction, the work reported here provides new insight into the immunological adaptation of cockroaches to life in septic environments as well as the factors that regulate bacterial pathogen transmission by these insects.

The results of our qRT-PCR analyses demonstrate that the AMP response in the gut of *B. germanica* is driven by a recognition process that is more specific than sensing of a general PAMP such as PGN. Most notably, the Blattellicin and two Attacin genes that we examined were both induced in response to the live wild-type strain of *S.* Typhimurium but not heat-killed *S.* Typhimurium or live *E. coli*, although the two are very similar Gram-negative enterobacteria. In contrast, in many insects, diverse live and heat-killed bacterial challenges induce AMP expression when ingested as a result of stimulation of the Toll and IMD pathways by conserved PAMPs. In the silkworm, ingestion of heat-killed *Pseudomonas aeruginosa* induces systemic AMP production (Miyashita et al., 2015), as does ingestion of live *Erwinia carotovora*, or heat-killed *Staphylococcus aureus* or *E. coli* by *D. melanogaster* larvae (Basset et al., 2000; Wen et al., 2019). Ingestion of heat-killed *E. coli* by *D*. *melanogaster* larvae can even result in upregulated AMP expression in young adult flies (Patrnogic et al., 2018). Further, in adult fruit flies, purified PGN from Gram-negative bacteria is sufficient to induce AMP expression in the gut without additional stimuli (Zaidman-Rémy et al., 2006).

The housefly, *Musca domestica*, is a comparable model for insect immunity that shares some of the ecological features of cockroaches. That is, houseflies similarly live and breed in septic environments, often consuming extremely high loads of diverse bacteria (Nayduch and Burrus, 2017). In the housefly gut, even though the AMP response to ingested *E. coli* was not found to be robust, expression of Diptericin was nonetheless induced after feeding (Fleming et al., 2014). Ingestion of *Campylobacter jejuni* (Gill et al., 2017) or *S. aureus* (Nayduch et al., 2013) similarly induces AMP expression in the gut. Moreover, *Pseudomonas aeruginosa* upregulates Defensin expression in the housefly gut when ingested (Joyner et al., 2013). Considering these published data, the observed lack of AMP response in the cockroach gut after ingesting high doses of bacterial PAMPs may be somewhat unique to cockroaches rather than a general adaptation to life in septic environments.

That AMP expression in the gut of *B. germanica* was not induced by the invAspiB mutant *S*. Typhimurium strain but was induced by the wild-type strain provides additional evidence that AMP expression is not robustly induced by conserved PAMP sensing, but rather by more specific bacterial colonization processes, such as type III secretion. This observation is in line with the finding that heat-killed *S*. Typhimurium did not induce any of the same responses induced by live bacteria. It is not likely that the induction of AMP expression by live wild-type *S.* Typhimurium is due to bacterial replication for several reasons. First, while *S.* Typhimurium does undergo replication in the cockroach gut (Turner et al., 2021), unlike *E. coli* (Zurek and Schal, 2004; Ray et al., 2020), a period of 1 h does not allow for significant expansion of the bacterial population. Second, the invAspiB mutant, which did not induce the same AMP responses as the wild-type strain, replicates to a similar degree as the wild-type strain (Turner et al., 2021).

The induction of AMP expression in the cockroach gut also does not appear to be tied to the pathogenesis of the ingested organism, as neither *E. coli* nor *S.* Typhimurium are pathogenic to cockroaches. However, AMP expression in response to ingested *S*. Typhimurium could be a result of cellular perturbations triggered by type III secretion effectors or a manipulation by this bacterium to increase its colonization and transmission (i.e. effector triggered immunity, Rajamuthiah and Mylonakis, 2014). Our previous work has demonstrated that the commensal gut microbiota hinders the persistence of *E*. *coli* in the cockroach gut (Ray et al., 2020). Thus, inducing AMP expression may alter the composition of the gut microbiota in a way that facilitates *S.* Typhimurium colonization. This hypothesis needs to be tested experimentally, but we have previously shown that type III secretion is required for efficient shedding of *S.* Typhimurium in cockroach feces (Turner et al., 2021), and in mammalian hosts type III secretion effectors can target NF-kB signaling to alter the immune response (Sun et al., 2016). Alternatively, the cockroach immune system may employ unknown mechanisms to fine tune the immune response by recognizing active colonization processes as signals of a potentially dangerous infection (Heil and Land, 2014). Future analysis of additional *S*. Typhimurium mutants could provide much insight into the specific type III secretion system and effectors underlying the phenomenon we describe.

The possible induction of Defensin expression only 24 h after ingestion of live, mutant, and heat-killed *S.* Typhimurium, though not statistically significant, is a curious observation. We are unable to explain this observation based solely on the available data, but it is possible that the effect is either an indirect result of commensal microbial community shifts that may occur following ingestion of a high load of bacteria, or a direct but delayed effect of lingering bacterial PAMPs.

**Fig. 4.**
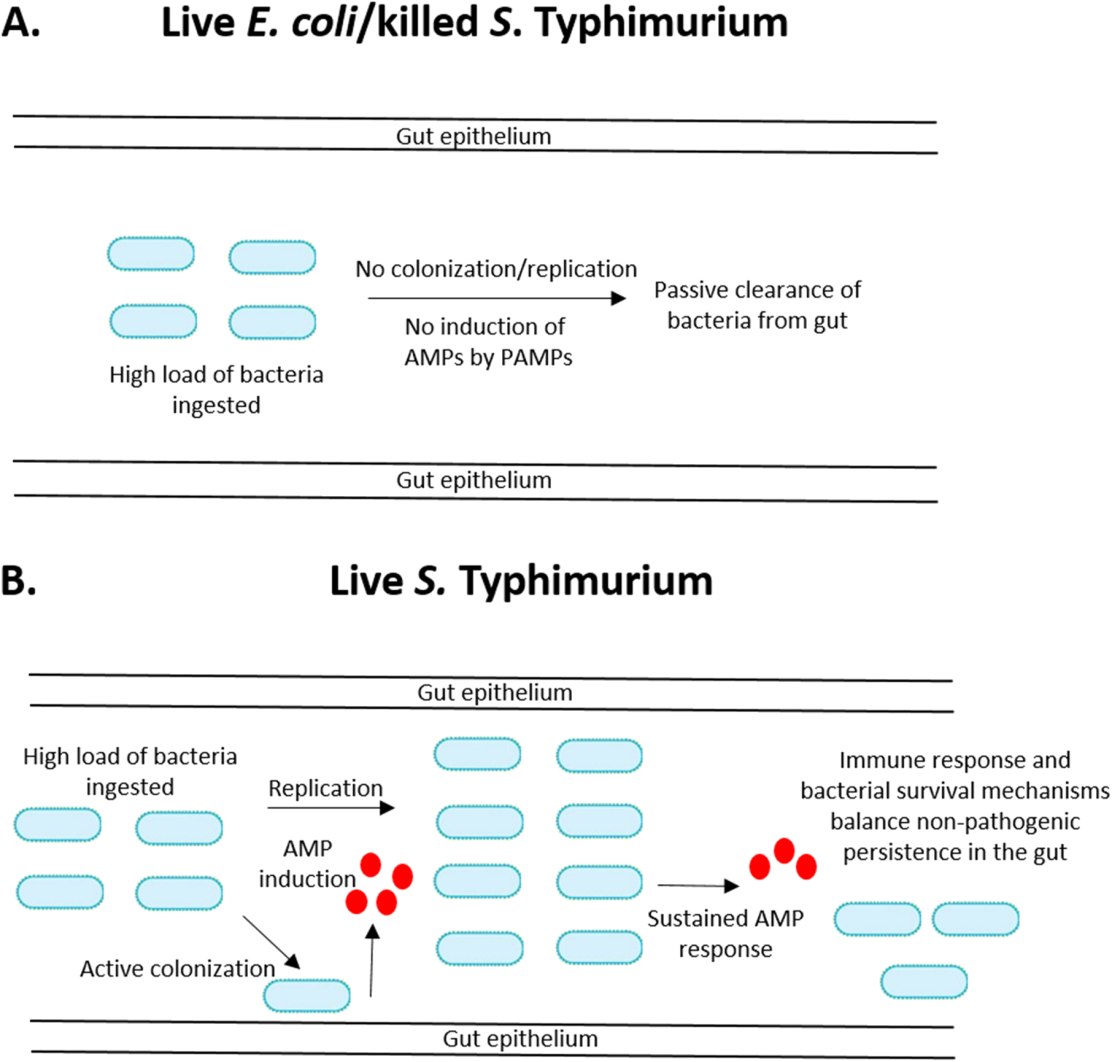
**Model of antimicrobial peptide response to enterobacterial infection in the cockroach gut.** This model incorporates insight from the present study and from our previous work analyzing the colonization dynamics of *S.* Typhimurium and *E. coli* in the cockroach gut (Ray et al., 2020; Turner et al., 2021). For additional details, see the Discussion section.

Based on the data reported here and our previous studies (Ray et al., 2020; Turner et al., 2021), we have developed a working model of the AMP response to enterobacterial infection in the cockroach gut (Fig. 4). Our data indicate that ingestion of high levels of bacterial PAMPs in the form of dead bacteria or live, non-colonizing bacteria that persist only transiently (e.g. *E. coli*) does not induce an AMP response (Fig. 4A). Instead, such bacteria are likely passively cleared via peristalsis or exclusion by the microbiota (Ray et al., 2020). Avoiding mounting an immune response to such frequent but innocuous stimuli could be a way to conserve energetic resources. On the other hand, ingested bacteria that actively colonize the cockroach gut (e.g. *S*. Typhimurium) trigger AMP expression as a result of more specific processes employed by these organisms, such as type III secretion (Fig. 4B). In such cases, the bacterial load may be stabilized at a non-pathogenic level by a balance between the immune response and bacterial replication and/or survival mechanisms (e.g. immune evasion).

Future studies are required to understand the molecular mechanisms underlying the phenomena we report here. Specifically, it remains completely unknown how the Toll and IMD pathways are regulated in the cockroach gut, how an expanded number of PGRP homologs contribute to innate immune signaling, and whether there exist additional pathways for regulating AMP expression. For example, negative feedback mechanisms similar to those involving PGRP-LB and PGRP-LF in the fruit fly could be mediated by unique cockroach PGRPs (Maillet et al., 2008; Paredes et al., 2011). In addition, it is possible that only a subset of the 39 cockroach AMPs may be induced by PAMPs. Lastly, some post-transcriptional regulation of AMP production could be at play in cockroaches. The latter phenomenon has been suggested in house flies in light of data demonstrating that Lysozyme gene expression in the gut is mostly constitutive and that AMP protein levels rather than mRNA are correlated with fluctuating bacterial densities during infection (Joyner et al., 2013; Nayduch and Joyner, 2013). RNAi is available in cockroaches and would be a useful approach to delineate the contributions of individual host genes of interest to the immune response.

Ultimately, our findings advance knowledge of the regulation of insect immunity and illuminate several interesting new mechanistic research directions. They also have implications for understanding pathogen transmission by cockroaches and for controlling infestations. The minimal AMP response to PAMPs in the gut may explain why cockroaches are adept at disseminating a diverse range of bacteria in a viable state in their feces even if those bacteria do not replicate. On the other hand, colonizing human pathogens such as *S*. Typhimurium may actively trigger the cockroach immune response and survive in spite of it as a result of replication and immune evasion. Thus, cockroach AMPs could be targets for transmission blocking interventions. The immune system could also be a potential target for the development of novel insecticides or biological control approaches that dysregulate interactions with symbionts or entomopathogens (Pan and Zhang, 2020).

## MATERIALS AND METHODS

### Cockroaches

The American Cyanamid Orlando laboratory strain of *B. germanica* was used in the present study, as in our previous work (Ray et al., 2020; Turner et al., 2021). Cockroach colonies were maintained in plastic enclosures at 25±1°C and 40–45% relative humidity on a 12:12 (L:D) hour photoperiod. The colonies were steadily provided dog chow (Purina, St. Louis, MO, USA) and tap water, and were given egg carton harborages for shelter. Adult males were used in experiments in order to preserve females for colony propagation and minimize physiological variation due to gonadotropic and developmental cycles.

### Bacterial strains and culture

The *S.* Typhimurium strain used in the present study was strain 14028. This bacterium replicates in cockroach gut, persisting for at least a week (Turner et al., 2021). To test the effects of *S*. Typhimurium type III secretion, strain SPN452, a type III secretion system 1/2 double mutant (invAspiB) derived from strain 14028 was used (Raffatellu et al., 2009). The *E. coli* strain used in the present study was strain B21, a derivative of *E. coli* K12 (Ward's Science, Rochester, NY, USA). This bacterium does not replicate in the cockroach gut and quickly declines after it is ingested (Ray et al., 2020). All strains were cultured in liquid LB medium at 37°C.

### Administration of bacteria to cockroaches

Bacterial cultures were provisioned orally. First, groups of adult male cockroaches were separated into experimental enclosures and starved of food and water for 3 days to promote consistent experimental feeding (Ray et al., 2020; Turner et al., 2021). Following the starvation period, a shallow Petri dish containing a stationary-phase culture of live or heat-killed bacteria diluted to OD_600_=1 was provided to the cockroaches as a sole food source for 30 min. This concentration results in an average ingested bacterial load of ∼3.56×10^6^ CFU per insect (Turner et al., 2021). Heat-killing of bacteria was carried out by incubation at 70°C for 2 h prior to feeding and was verified by lack of growth on LB agar plates. Cockroaches fed sterile LB medium served as controls for baseline expression in all experiments. Blue food dye was added to the cultures to enable tracking of fed cockroaches and unfed cockroaches were excluded. Immediately after the feeding period, the bacterial culture was removed, and dog chow and water were provided. Cockroaches were collected for gene expression analysis 1 and 24 h after feeding. Our previous work determined that between 0- and 6-h post-ingestion, *S*. Typhimurium undergoes a ∼tenfold expansion in the gut of *B. germanica*, while between 6- and 24-hours post-ingestion, the bacterium undergoes a ∼1000-fold bottleneck (Turner et al., 2021).

### Survivorship of cockroaches fed *S.* Typhimurium

We previously determined that the *E. coli* B21 strain used in this study is not pathogenic to cockroaches when ingested (Ray et al., 2020). To determine whether *S.* Typhimurium is pathogenic to cockroaches when ingested, survivorship was compared to control cockroaches fed sterile LB medium. The number of deaths occurring in control and *S.* Typhimurium-fed cohorts was monitored periodically over a period of 20 days. Two independent trials were conducted including a total of 41–44 cockroaches per treatment. For statistical analysis, a Fisher's exact test was used to compare the proportion of insects surviving at the end of the experimental period.

### Design of primers for qRT-PCR

qRT-PCR primers were designed for five selected AMP genes: Attacin 1, Attacin 2, Blattellicin 1, Defensin 1, and Defensin 2 (Table 1). The sequences of these genes were obtained from a recently published *in silico* study (Silva et al., 2020). Primer sequences were designed using Primer3 software and specificity was evaluated *in silico* using Primer-BLAST (Untergasser et al., 2012). Custom oligonucleotides were synthesized by MilliporeSigma (Burlington, MA, USA). To further verify specificity, melt curve analysis was performed after amplification of cockroach cDNA with each primer set. Due to the extremely high sequence similarity between Defensin 1 and Defensin 2 genes, it was not possible to design primers that were specific for each. Instead, we designed a primer set that targeted both genes simultaneously.

**Table 1.**
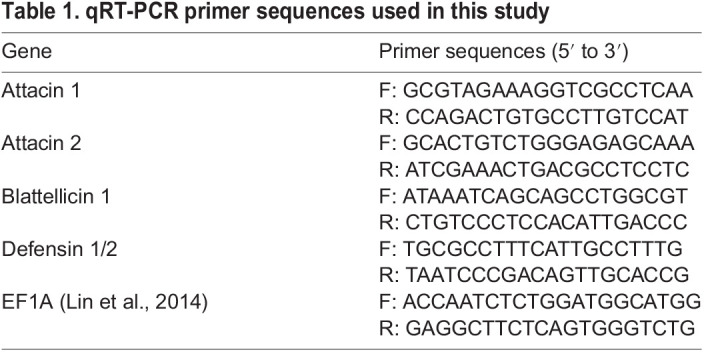
qRT-PCR primer sequences used in this study

### qRT-PCR analysis of antimicrobial peptide gene expression

AMP gene expression was evaluated in the guts of four to five individual cockroaches per treatment per time point. In brief, 1-or-24 h after feeding on bacterial cultures, whole guts (foregut, midgut, hindgut) were dissected under a stereomicroscope. RNA was isolated from individual guts using TRIzol reagent (Thermo Fisher Scientific, Waltham, MA, USA) according to the manufacturer's protocol. RNA samples were treated with DNase I (Thermo Fisher Scientific) to remove contaminating traces of genomic DNA. Subsequently, the RNA concentration in each sample was determined using a Qubit fluorometer (Thermo Fisher Scientific) and RNA was converted to cDNA using the high capacity cDNA reverse transcription kit (Applied Biosystems, Waltham, MA, USA). qRT-PCR was performed on a QuantStudio 3 instrument (Applied Biosystems) using the PowerUp SYBR Green Master Mix (Applied Biosystems) with primers at a concentration of 500 nM. The amplification conditions were set to the instrument default for a fast run as follows: 95°C for 20 s, 40 cycles of 1 s at 95°C and 20 s at 60°C. Triplicate reactions were run for each sample and gene target and cycle threshold (CT) values were averaged. Each run included negative control reactions with no template.

From CT values, expression of each AMP gene was calculated relative to the common housekeeping gene, EF1A (Lin et al., 2014; Ray et al., 2020; Zhu et al., 2021), using the delta-CT method. In instances where expression of a particular AMP gene was not detectable (e.g. Blattellicin 1 expression in several unstimulated control guts), the CT value was conservatively set to 40 to enable quantitative analysis. Outlier relative expression values were identified using ROUT testing and excluded from the final statistical analysis. Statistical analysis to determine if AMP gene expression was significantly induced in cockroaches that had ingested bacteria relative to baseline expression (unstimulated controls) consisted of a Kruskal–Wallis one-way ANOVA or an unpaired *t*-test as appropriate. *P*-values <0.05 were considered statistically significant.
